# Cognitive functioning and sustained internet use amid the COVID-19 pandemic: longitudinal evidence from older adults in Switzerland

**DOI:** 10.1038/s41598-024-69631-w

**Published:** 2024-08-13

**Authors:** Maud Wieczorek, Robert Reinecke, Carmen Borrat-Besson, Clément Meier, Maximilian Haas, Andreas Ihle, Matthias Kliegel, Jürgen Maurer

**Affiliations:** 1 Swiss Center of Expertise in Life Course Research LIVES, Lausanne and Geneva, Switzerland; 2 Faculty of Business and Economics (HEC), University of Lausanne, Lausanne, Switzerland; 3 Swiss Centre of Expertise in the Social Sciences (FORS), University of Lausanne, Lausanne, Switzerland; 4 Faculty of Biology and Medicine (FBM), University of Lausanne, Lausanne, Switzerland; 5 Department of Psychology, University of Geneva, Geneva, Switzerland; 6 Centre for the Interdisciplinary Study of Gerontology and Vulnerability, University of Geneva, Geneva, Switzerland; 7 UniDistance Suisse, Brig, Switzerland

**Keywords:** Psychology, Cognitive ageing

## Abstract

This study aimed to investigate the relationship between pre-pandemic objective and subjective cognitive functioning and sustained Internet use during the pandemic among older adults in Switzerland. Data from 1299 respondents of the Survey of Health, Ageing and Retirement in Europe (SHARE) in 2019/2020 and a supplementary technology use questionnaire during the pandemic in 2021 were used. Cognitive functioning was assessed in 2019/2020 through objective measures (delayed and immediate memory, verbal fluency) and self-rated memory. Sustained Internet use was defined as having used the Internet at least once in the past seven days in 2019/2020 and reporting daily or weekly use in 2021. We found that 73.1% of respondents consistently used Internet between 2019/2020 and 2021. Using multivariable probit regression models controlling for sociodemographic and health variables, we found that higher global cognition z-scores, especially in immediate and delayed memory, were associated with a higher likelihood of sustained Internet use. Additionally, respondents with good, very good, or excellent self-rated memory were more likely to sustain their Internet use. These findings highlight the potential critical role of cognitive health in shaping older adults’ digital engagement, suggesting that cognitive assessments and training should be further considered in digital literacy initiatives for this population.

## Introduction

The advent of digital technology has transformed society, becoming a cornerstone in public, economic, and social spheres and reshaping the way we communicate, access information and services. Recent studies have highlighted the positive impact of information and communication technologies on lifestyle behaviors, as well as on the physical and mental health outcomes of older adults^1–4^. However, to fully reap these benefits, it is essential for the older population to use these technologies.

In the past decade, research has described the “grey digital divide”, a phenomenon referring to the unequal access to and limited use of the Internet among older adults, as compared to younger adults^5^. The COVID-19 pandemic has intensified this divide, as social restrictions have made the online world an essential component for maintaining social interaction (e.g., instant messaging, email, video calls, social networking), leisure activities (e.g., gaming and browsing information), and the execution of daily tasks (e.g., banking, shopping, paying bills)^6,7^. This pandemic has highlighted the intersection between digital and social inequalities, where digitally excluded individuals face more challenges in adapting to rapid social changes^8,9^. Therefore, identifying factors that facilitate or hinder the adoption or the sustained use of the Internet in later life is critical to inform interventions and policies aimed at bridging these digital gaps between generations.

While the outcomes and barriers of technology use in older age have been largely discussed^10–12^, the determinants of the sustained use of the Internet over time are less explored^13,14^. Effective and continuous use of the Internet may depend not only on prerequisite skills but also on various socio-demographic and health characteristics; one of them being good cognitive functioning.

Cognitive functioning involves a range of mental processes related to the acquisition, storage, manipulation, and retrieval of information, all essential for effectively navigating and utilizing digital technologies. A substantial body of evidence has examined the role of Internet in shaping cognitive functioning^15–21^ and several cross-sectional studies have examined the relationship between cognitive functioning and Internet use in older adults across different populations and contexts^22–25^. However, large longitudinal studies investigating this relationship are more limited. Earlier studies using data collected in the early 2000’s and 2010’s from the US Health and Retirement Study^26^, the Survey of Health, Ageing and Retirement in Europe (SHARE)^19^, the China Health and Retirement Longitudinal Study^27^ and the German Ageing Survey^28^, have reported a positive association between higher cognitive functioning and the Internet use. Yet, Berner et al. presented mixed results, indicating that higher cognitive functioning was associated with a higher likelihood of both stopping and starting to use the Internet over 10 years^29^. Notably, these large longitudinal studies have not considered subjective cognitive functioning, i.e. appraisal of one’s own memory, in relation with Internet use. This oversight is significant, as an individual’s perception of their cognitive abilities may influence their willingness to engage with new technologies^30^. Older adults with a self-perceived decline in cognitive functions might have low self-efficacy^31^ (i.e. a particular set of beliefs that determine how well one can execute a plan of action in prospective situations^32^) and therefore might feel less capable of learning or using new technologies, regardless of their actual cognitive abilities to do so.

Given the limited and conflicting evidence on the link between subjective and objective cognitive functioning and Internet use, along with the lack of recent data on the determinants of Internet use patterns during the COVID-19 pandemic, our novel scientific contribution is threefold. First, our study aimed to investigate the relationship between pre-pandemic objective and subjective cognitive functioning and sustained use of the Internet during the pandemic in a large population-based sample of older adults in Switzerland. Second, we explored in detail how pre-pandemic objective and subjective cognitive functioning were associated with changes in the frequency of six specific Internet-based activities that appeared to have played a pivotal role during the pandemic (sending or receiving emails, having video calls, chatting, purchasing goods or services, searching for health information and searching for general information). Third, we conducted separate analyses for four key cognitive everyday-relevant abilities (immediate and delayed memory, verbal fluency, and self-rated memory) to account for differences in their respective associations with Internet use over time.

## Results

### Main characteristics of the analytical sample

The analytical sample’s characteristics are detailed in Table 1. Over half of the respondents were female (53.4%), and nearly half (43.7%) were aged 65–74 years. A large majority held a medium level of education (64.1%) and 56.8% and 31.3% of respondents could easily or fairly easily make their ends meet, respectively. Most of the respondents lived in the German-speaking regions of Switzerland (73.8%) and in rural areas (54.3%). In terms of health, 42.3% of respondents categorized their health as good and 41.3% as very good or excellent, while 84.7% reported no depressive symptoms. Also, 81.9% of respondents rated their current memory performance as either good, very good, or excellent.

**Table 1:** Main characteristics of the analytical sample, adults aged 58 + , Survey of Health, Ageing, and Retirement in Europe—Switzerland, 2019/2020, n = 1299.

		Unweighted	
		n	%
Sex	Men	605	46.6
	Women	694	53.4
Age groups	58–64 years	318	24.5
	65–74 years	567	43.7
	75 + years	414	31.9
Education levels	Low	203	15.6
	Medium	833	64.1
	High	263	20.3
Partnership status	Has a partner	989	76.1
	No partner	310	23.9
Make ends meet	Easily	738	56.8
	Fairly easily	407	31.3
	With difficulty	154	11.9
Swiss linguistic regions	German	959	73.8
	French	305	23.5
	Italian	35	2.7
Living area	Urban	593	45.7
	Rural	706	54.3
Self-rated health	Poor/Fair	212	16.3
	Good	550	42.3
	Very good/Excellent	537	41.3
Depressive symptoms	Yes	199	15.3
	No	1100	84.7
Self-rated memory	Poor/Fair	235	18.1
	(Very) good/Excellent	1064	81.9

*CI* confidence intervals.

**Figure 1:**
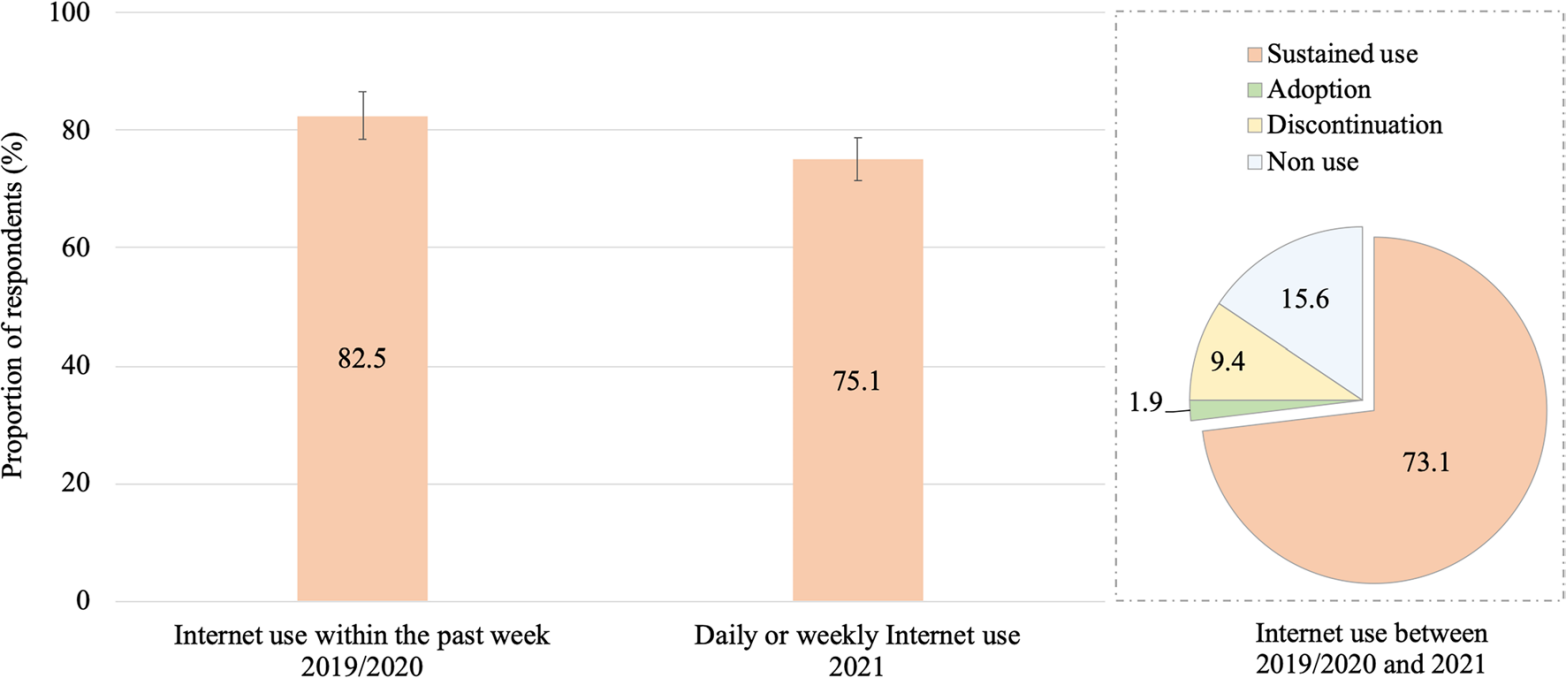
Patterns of Internet use between 2019/2020 and 2021, adults aged 58 + , Survey of Health, Ageing, and Retirement in Europe—Switzerland, 2019/2020–2021, n = 1299.

Figure 1 presents the different patterns of Internet use. In 2019/2020, 82.5% of the respondents reported using the Internet within the past week, a figure that changed to 75.1% for daily or weekly usage in 2021. Overall, 73.1% of respondents reported regular Internet use in both 2019/2020 and 2021. Conversely, a small proportion (1.9%) of the respondents only started using the Internet during this period, and 9.4% stopped doing so. Additionally, 15.6% reported not using the Internet during these two years.

### Cognitive functioning and Internet use in 2019/2020

Table 2 presents the partial associations between cognitive functioning and Internet use from adjusted multivariable models. When controlling for sex, age groups, education levels, partnership status, subjective financial situation, living area, linguistic region, self-rated health and depressive symptoms, we found that higher global cognition z-scores were significantly associated with a higher likelihood of using the Internet (Model 1: APE = 0.09, p < 0.001).

**Table 2:** Partial associations between cognitive functioning and Internet use in 2019/2020, adults aged 58 + , Survey of Health, Ageing, and Retirement in Europe—Switzerland, n = 1299.

	Internet use in 2019/2020				
	Model 1	Model 2	Model 3	Model 4	Model 5
Global cognition,	0.09***			0.08***	
z-score	**(0.01)**			**(0.01)**	
Immediate memory,		0.03*			0.03
z-score		**(0.01)**			(0.01)
Delayed memory,		0.04**			0.04**
z-score		**(0.01)**			**(0.01)**
Verbal fluency,		0.02			0.01
z-score		(0.01)			(0.01)
Self-rated memory,					
Good, very good or excellent (vs poor or fair)			0.10*** (0.03)	0.07** (0.03)	0.07** (0.03)

The table shows average partial effects and standard errors in parentheses from separate models for cognitive functioning and Internet use.

Statistical significance:*p < 0.05, **p < 0.01, ***p < 0.001.

The probit regression models control for sex, age groups, education levels, partnership status, subjective financial situation, living area, linguistic region, self-rated health and depressive symptoms.

Significant values are bold.

In the model including all three cognitive abilities, both higher immediate and delayed memory z-scores were significantly associated with an increased probability of using the Internet (Model 2: APE = 0.03, p < 0.05 and APE = 0.04, p < 0.01, respectively). The association between verbal fluency z-scores and the Internet use, however, was not statistically significant.

When considering self-rated memory as the main independent variable, we found that respondents who reported good, very good or excellent memory performance were significantly more likely to use the Internet, compared to their counterparts with fair or poor self-rated memory (Model 3: APE = 0.10, p < 0.001). The overall significance and directions of the associations between the global cognition z-score, self-rated memory and Internet use remained consistent across models including the objective and subjective cognitive variables (Models 4 and 5).

### Cognitive functioning and sustained use of Internet between 2019/2020 and 2021

The partial associations between cognitive functioning and the sustained use of the Internet between 2019/2020 and 2021 from adjusted multivariable models are presented in Table 3.

**Table 3:** Partial associations between cognitive functioning and Internet use between 2019/2020 and 2021, adults aged 58 + , Survey of Health, Ageing, and Retirement in Europe—Switzerland, n = 1299.

	Sustained use of the Internet between 2019/2020 and 2021				
	Model 1	Model 2	Model 3	Model 4	Model 5
Global cognition,	0.12***			0.12***	
z-score	**(0.02)**			**(0.02)**	
Immediate memory,		0.05**			0.05**
z-score		**(0.02)**			**(0.02)**
Delayed memory,		0.05***			0.05**
z-score		**(0.02)**			**(0.02)**
Verbal fluency,		0.02			0.02
z-score		(0.01)			(0.01)
Self-rated memory,					
Good, very good or excellent (vs poor or fair)			0.11*** (0.03)	0.07* (0.03)	0.07* (0.03)

The table shows average partial effects and standard errors in parentheses from separate models for cognitive functioning and Internet use.

Statistical significance:*p < 0.05, **p < 0.01, ***p < 0.001.

The probit regression models control for sex, age groups, education levels, partnership status, subjective financial situation, living area, linguistic region, self-rated health and depressive symptoms.

Significant values are bold.

After accounting for key sociodemographic and health-related variables, respondents with higher global cognition z-scores were significantly more likely to have continuously used the Internet between 2019/2020 and 2021 (Model 1: APE = 0.12, p < 0.001). Consistent with our previous analyses, a significant association was found between higher immediate and delayed memory z-scores and a greater probability of maintaining the Internet use over the same period (Model 2: APE = 0.05, p < 0.01 and APE = 0.05, p < 0.001, respectively). Nonetheless, there was no significant association between verbal fluency z-scores and maintenance of the Internet use between the two time points. In a final model, global cognition z-scores remained significantly and positively associated with maintaining the Internet use (Model 4: APE = 0.12, p < 0.001) while good, very good or excellent self-rated memory remained positively associated with the sustained use of the Internet between 2019/2020 and 2021 (APE = 0.07, p < 0.05).

### Cognitive functioning and frequency of Internet-based activities during the COVID-19 pandemic

**Figure 2:**
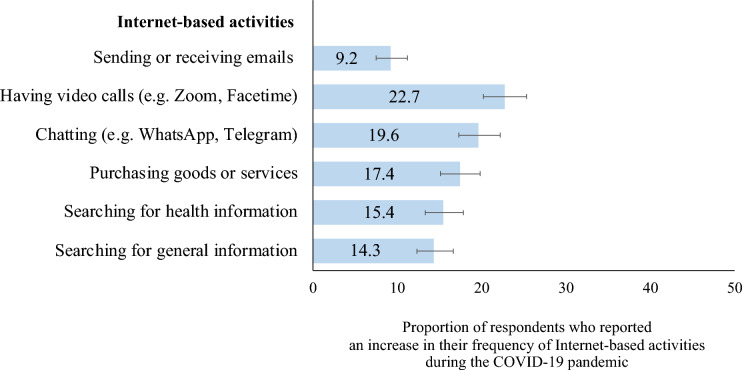
Proportion of respondents who reported an increase in the frequency of Internet-based activities during the COVID-19 pandemic along with 95% confidence intervals, adults aged 58 + , Survey of Health, Ageing, and Retirement in Europe—Switzerland, 2019/2020–2021, n = 1019.

Figure 2 shows the proportions of respondents who reported an increase in the frequency of different Internet-based activities during the COVID-19 pandemic. Overall, 22.7% of respondents reported an increase in the frequency of using the Internet to have video calls during the COVID-19 pandemic, compared to their pre-pandemic use. Similarly, 19.6% reported using the Internet more often for chatting during the pandemic, compared to their pre-pandemic usage. In our exploratory multivariable analyses, respondents with higher global cognition z-scores were more likely to have used the Internet more frequently to have video calls (APE = 0.08, p < 0.001) and for chatting (APE = 0.03, p < 0.05) during the COVID-19 pandemic (Table 4). Additionally, respondents who rated their memory as good, very good or excellent were significantly less likely to have used the Internet for video calls more often during the pandemic, compared to those who rated their memory as poor or fair (APE = − 0.09, p < 0.05). We did not find a significant association between global cognition z-scores, self-rated memory and the change in frequency of other Internet-based activities during the COVID-19 pandemic.

**Table 4:** Partial associations between cognitive functioning and change in the frequency of Internet-based activities during the COVID-19 pandemic, adults aged 58 + , Survey of Health, Ageing, and Retirement in Europe—Switzerland, 2019/2020–2021, n = 1019.

	Sending or receiving emails	Having video calls	Chatting	Purchasing goods or services	Searching for health information	Searching for general information
Global cognition,	0.02	0.08***	0.03*	0.02	0.01	**− **0.01
z-score	(0.01)	**(0.02)**	**(0.02)**	(0.02)	(0.02)	(0.02)
Self-rated memory,						
Good, very good or excellent (vs poor or fair)	-0.02 (0.03)	− 0.09* (0.04)	0.01 (0.04)	**− **0.06 (0.04)	0.00 (0.03)	0.04 (0.03)

The table shows average partial effects and standard errors in parentheses from separate models for cognitive functioning and Internet use.

Statistical significance:*p < 0.05, **p < 0.01, ***p < 0.001.

The probit regression models control for sex, age groups, education levels, partnership status, subjective financial situation, living area, linguistic region, self-rated health and depressive symptoms.

Significant values are bold.

## Discussion

Our study, analyzing data from 1,299 adults aged 58 and older in the general population, offers novel and important insights into the association between pre-pandemic cognitive functioning and sustained Internet use during the COVID-19 pandemic. In the context of Switzerland, characterized by a high internet penetration rate^33^ and a significant aging population^34^, our study reveals how a digitally connected and aging society adapts to increased reliance on technology in extraordinary times. We found that 73.1% of respondents consistently used the Internet both before and during the COVID-19 pandemic, indicating a significant level of digital engagement in this population group. Independently of key socio-demographic and health characteristics, we found that respondents with higher global cognition scores, particularly in the domains of immediate and delayed memory, were significantly more likely to have continuously used the Internet between 2019/2020 and 2021.

These findings extend the previous literature in several ways. In a longitudinal population-based study in Finland, Heponiemi et al. reported that word list immediate and delayed recall were both significantly associated with non-use of the internet for services three years later^35^. However, the authors only controlled for age and sex in their models, which increases the risk for residual confounding. This limitation is particularly significant as additional characteristics such as education, socio-economic status, and health-related variables can also influence cognitive functioning or the use of Internet^26,36^. Our study addresses this gap by incorporating a broader range of covariables, thereby offering more comprehensive insights and enhancing the robustness of our findings. In another study using data from the Dutch Longitudinal Aging Study Amsterdam, Berner et al. assessed the relationship between cognitive functioning measured with the Mini-Mental State Examination and starting or stopping the use of the Internet over 10 years^29^. The authors reported a positive association between respondents’ cognitive abilities and the likelihood of starting and stopping Internet usage, highlighting that individuals with better cognitive abilities were significantly more likely to both adopting and discontinuing Internet use. However, the authors acknowledged that the conduct of the data collection between 2000 and 2010—before the appearance of new devices such as tablet computers or smartphones—is a significant limitation of their study.

While current evidence seems suggesting a positive and significant association between overall cognitive functioning and Internet use over time, the heterogeneity in the measurement of cognitive functioning and the outcome definitions across studies precludes direct comparison. Unlike the above-mentioned studies, the present study used an age- and education-specific global cognition z-score, calculated as the average of the z-scores of immediate memory, delayed memory, and verbal fluency, offering a comprehensive assessment of cognitive abilities. This method allows capturing the multidimensionality of cognitive functioning and by adjusting for age and education, provides a more accurate reflection of cognitive performance while accounting for potential demographic differences. This approach also addresses the limitations of floor and ceiling effects often observed in some cognitive tests^37,38^.

Building the z-scores of immediate memory, delayed memory, and verbal fluency enabled us to explore in detail the respective associations between each individual cognitive ability and the sustained use of the Internet. Our study revealed a significant association between higher immediate and delayed memory z-scores and a greater probability of maintaining the Internet use between 2019/2020 and 2021. Conversely, we found no significant association between verbal fluency z-scores and sustained Internet use during this period, aligning with prior cross-sectional studies that suggest varying associations between different cognitive abilities and technology use. Memory, in particular, has been found to be associated with technology skills and use^22^, while verbal abilities have been shown to have no or weak association with information and communication technology use^39,40^. The lack of a significant association between verbal fluency and sustained Internet use may be due to the complex cognitive processes required for using technology. These processes may extend beyond performances on verbal fluency tasks which require speed, attention, self-monitoring, and inhibition^41^, to encompass other aspects such as visual-spatial and problem-solving skills^42^. Similarly, this multifaceted cognitive demand may also explain why we did not observe significant associations between overall cognitive functioning z-scores and changes in the frequency of specific Internet-based activities. For instance, activities like purchasing goods or services, searching for health or general information may entail more complex cognitive processes than simpler tasks like having video calls or chatting. These intricate processes might not have been fully captured by our measure of overall cognitive functioning. Therefore, further large-scale longitudinal studies are still needed to confirm our findings in independent study populations of older adults and thoroughly understand these complex relationships.

Our additional focus on self-rated memory further contributes to the nuanced understanding of the relationship between cognitive functioning and Internet use. Contrasting with a cross-sectional Finnish study among older migrants, which did not find a significant association between self-rated memory and difficulties in using the Internet^43^, our study presents a different context. Our findings suggest a consistent positive and significant relationship between self-rated memory and sustained Internet use, even after accounting for objective cognitive functioning. Notably, the above-mentioned Finnish postal survey targeted a specific migrant, Russian-speaking population and focused on qualitative aspects of Internet challenges (e.g., perceptions that the Internet is too complicated or hard to learn). In contrast, our study assessed the frequency of Internet use in a broader, population-based sample of older adults living in Switzerland. Importantly, individuals with limited national language skills are not eligible to participate in the Swiss component of SHARE, ensuring that participants have sufficient language proficiency, which might influence their Internet use and cognitive assessments. These methodological and demographic differences likely contribute to the variations in findings between the two studies. The consistent positive and significant relationship between self-rated memory and sustained Internet use we are reporting may be partly explained by the link between self-efficacy and processes influencing memory performance^44^. In this sense, lower self-efficacy may likely lead to lower control and regulation of affective, cognitive, and motivational processes related to self-appraisal of memory function either by being associated with more negative appraisals of memory or by amplifying the effect of negative emotional responses on memory performance^44^. Berner and colleagues have notably reported that psychological variables such as sense of mastery, self-esteem, and self-efficacy were strongly associated with starting or stopping using the Internet^29^. Additionally, the current generation of older individuals may have low self-efficacy in domains related to modern technology such as the Internet and computers, because they lacked substantial learning and training experiences with these technologies earlier in life^45,46^. The findings of this study therefore underscore the importance of both objective and subjective cognitive functioning in shaping older adults’ digital engagement.

Previous research indicated that digital literacy programs can empower older persons, foster social participation, and increase older adults’ autonomy. Tailored peer- or intergenerational training initiatives targeted at older individuals have proven to be effective in enhancing and maintaining their digital literacy^47–49^. Further, Martinez Alcala et al. have introduced several key principles for the older adults’ digital literacy training. They notably highlighted that the knowledge provided to older adults has to be useful to learn and it has to respond to their personal and social needs^50^, that may include cognitive functioning. Wolfson et al. have suggested that technology-based instruction for older adults should include information on cognitive and metacognitive strategies to facilitate learning (e.g. paraphrasing key ideas and creating links with existing knowledge or reflecting on their understanding of the material, generating and answering questions about it, and applying learned strategies to new, analogous problems)^51^. As further research is needed in this field^52,53^, policy makers and healthcare providers may consider cognitive assessments and training programs as part of future digital literacy initiatives aimed at older adults. These efforts have the potential to not only improve digital literacy but also enhance the participants’ self-efficacy in maintaining their technology use, aligning with findings that associate better cognitive self-assessment with increased digital engagement. At a broader policy level, initiatives should not only focus on making digital technologies accessible, affordable, and available across populations but also include tailored support for using everyday services that are transitioning online^53^. This includes facilitating access to government information and services, tele-health services and other digital platforms, thereby ensuring equitable access to essential goods and services that rely on digital technology^53^. Additionally, involving older adults, including those with disabilities, in the design process of digital technologies has the potential to improve both their user-friendliness and relevance. By considering the needs and preferences of older adults, digital solutions can be more effectively tailored to meet their requirements, ultimately leading to more inclusive and effective digital environments^53^.

Although our study offers valuable new insights, several limitations warrant consideration. First, while our study relies on data from a population-based survey with a high response rate, there remains a risk of residual selection biases. Specifically, certain subpopulations, such as the oldest-old adults and individuals with severe health or cognitive impairments, might be underrepresented in the SHARE respondents. This underrepresentation could skew the generalizability of our findings, particularly in relation to these groups. Second, the methods used to assess Internet use varied across survey waves. In 2019/2020, Internet use was assessed by a simple yes/no response for usage within the past week, whereas in 2021, usage frequency was detailed over the past six months. This change may have limited the full comparability of data across the two periods, potentially affecting the interpretation of the observed decline in Internet use. Third, the high prevalence of Internet use in 2019/2020 limited the sample size for modeling the different patterns of Internet use, including Internet adoption or discontinuation, which could have provided a more nuanced understanding of these dynamics, highlighting an area for future research. Additionally, our exploratory analysis did not include adjustment for multiple testing. This absence of correction means that the likelihood of false positives might be higher, necessitating a cautious interpretation of our findings, especially those that are marginally significant. Lastly, the observational nature of our study precludes inferring causality from the findings. Despite adjustment on several potential confounders, the associations observed might be influenced by unmeasured confounding variables.

In conclusion, the present study reported that a majority of older adults in Switzerland maintained consistent Internet use both prior to and during the COVID-19 pandemic. We found a consistent association between pre-pandemic higher global cognition scores, particularly in the immediate and delayed memory domains, and the likelihood of sustained Internet use during the pandemic. Also, older adults reporting good, very good, or excellent self-rated memory were more likely to maintain their Internet use between 2019/2020 and 2021. These results suggest the important role that cognitive functioning, both in objective assessments and self-perception, plays in shaping older adults’ digital engagement. In light of these findings, future initiatives in digital literacy for older adults could greatly benefit from incorporating cognitive assessments and training, paving the way for more inclusive and effective technology use particularly during disruptive events like pandemics.

## Methods

### Study design and participants

We used data from SHARE, a multidisciplinary and longitudinal population-based survey of older adults aged 50 and older across 28 European countries and Israel^54^. At each biennial wave, data on health, socioeconomic status, social, family networks, and other life circumstances were collected using internationally harmonised, face-to-face computer-assisted personal interviews, conducted by trained interviewers. The present study used data from the eighth wave of SHARE Switzerland, complemented by responses from a supplementary technology use questionnaire distributed during the COVID-19 pandemic in 2021. In total, 2009 older adults living in Switzerland and their partners participated in the face-to-face interviews as part of the eighth wave of SHARE Switzerland, which took place between October 2019 and March 2020^55,56^. As part of the second SHARE Corona Survey^57,58^, SHARE respondents in Switzerland were invited to complete a country-specific paper-and-pencil questionnaire between June and July 2021 which assessed their usage of and attitudes towards information and communication technologies.

At the time of sampling, SHARE Switzerland was designed to be nationally representative of community-dwelling individuals aged 50 and over. The last sample refreshment of the Swiss sample took place in 2011 to maintain its representativeness. By 2019/2020, individuals aged 50 to 58 could only enter SHARE as partners of target respondents, not as primary sampled individuals. Therefore, these survey participants were not representative of the general population aged 50–58. For this reason, the present study only included respondents, or their partners, aged 58 years and over in 2019/2020. After excluding 391 respondents who participated in the interview in 2019/2020 but did not complete the paper-and-pencil questionnaire on information and communication technologies in 2021, 36 respondents younger than 58 years old in 2019/2020, and 283 respondents with one or more missing answers on the outcome, exposure variables, or covariates, the final analytical sample consisted of 1299 individuals for the main analysis. Due to missing data on changes in frequency of specific Internet-based activities, we further restricted our analytical sample to 1019 respondents in our exploratory analysis.

### Outcomes

To assess the prevalence of Internet use in 2019/2020, participants were asked if they had used the Internet for emailing, searching for information, making purchases, or any other purpose at least once in the past seven days. Responses were binary: “yes” or “no”. In 2021, amidst the COVID-19 pandemic, participants were asked to report the frequency of their Internet use on average over the past six months, with response categories ranging from “every day” to “never”. We considered that the respondents had a regular Internet usage behavior if they had reported using the Internet every day or several times a week. For sample size considerations, the main outcome of the present study was the sustained use of the Internet between 2019/2020 and 2021, defined as having used the Internet at least once in the past seven days in 2019/2020 and reporting daily or weekly use in 2021. This binary outcome was categorized as “yes” for sustained use, and “no” for adoption, discontinuation or non-use of the Internet over the two time points. Additionally, we considered the change in the frequency of six Internet-based activities during the COVID-19 pandemic as exploratory outcomes. More specifically, the participants were asked to compare their current frequency of using the Internet to send or receive emails, have video calls, chat, purchase goods or services, search for health information and search for general information to their habits before the pandemic started.

### Main exposures

Cognitive functioning was assessed during the wave 8 computer-assisted personal interviews conducted in 2019/2020. For the present study, we considered three key cognitive abilities that were objectively measured, namely immediate memory, delayed memory, and verbal fluency. Immediate and delayed memory abilities were evaluated using the modified version of Rey’s Auditory Verbal Learning Test^59^, which entails immediate recall and delayed recall of a 10-word list. Verbal fluency was measured by the number of distinct animals a participant could name within one minute. For each of these three cognitive abilities, a higher score reflects better cognitive functioning. To account for individual variability^36^, adjustments based on age and education levels were applied to the cognitive scores. Age was grouped into three categories (58–64, 65–74, and 75 + years), and education was classified as low, middle, and high based on the International Standard Classification of Education (ISCED) of 2017^60^. The mean and standard deviation (SD) for immediate memory, delayed memory and verbal fluency were computed within each of the nine categories that resulted from the cross-tabulation of age and education. The raw score for each cognitive ability was transformed into a z-score by subtracting the mean and dividing by the SD corresponding to each age-education category. A global cognitive score was then computed by averaging the z-scores of all three cognitive abilities, with a higher z-score reflecting better cognitive functioning. In addition to these objective cognitive measures, self-rated memory was also assessed. Respondents were asked to rate their memory performance at the present time on a five-point scale ranging from “excellent” to “poor”. These responses were dichotomised as “good, very good or excellent” and “poor or fair”.

### Covariates

The covariates considered in the present study were socio-demographic variables, including sex (men, women) and age group (58–64 years, 65–74 years, 75 + years). Education levels were grouped into three categories based on the ISCED 2017 (low, medium, high)^60^. The binary variable for partnership status (has a partner, has no partner) considered all types of partnership based on the marital status and living arrangements of the respondents. The subjective financial situation of respondents was assessed based on the question: “Is your household able to make ends meet?”. The response categories were recoded as “easily”, “fairly easily”, and “with difficulty”. The variable related to respondents’ living area was dichotomised (urban, rural). The language used to answer the questionnaire (German, French, Italian) was used as a proxy for regional/cultural differences. We additionally considered two health characteristics as covariates: self-rated health, categorized into three levels—poor or fair, good, and very good or excellent; and the presence of depressive symptoms, assessed using the EURO-D depression scale, categorized as yes (a score of 4 or higher) or no (a score below 4)^61^.

### Statistical analysis

The characteristics of the analytical sample were described using number counts and corresponding proportions. The partial associations between the global cognition z-score, self-rated memory and sustained Internet use between 2019/2020 and 2021 were examined using probit regression models. The immediate memory z-score, delayed memory z-score and verbal fluency z-score were additionally entered in one unique model to investigate the association between these three cognitive abilities and the main outcome. Similarly, probit regression models were used to assess the partial associations between the global cognition z-score, self-rated memory and the change in frequency of Internet-based activities during the COVID-19 pandemic. The multivariable models accounted for sex, age groups, education levels, partnership status, subjective financial situation, living area, linguistic region, self-rated health and depressive symptoms. Results were reported as average partial effects (APE) along with corresponding standard errors (SE). Considering the potential for unobserved dependencies between observations, as both respondents and their partners could be part of the SHARE survey, we clustered the estimated standard errors at the household level within our multivariable models. Statistical analyses were conducted using STATA/SE 17.0 (STATA Corporation, College Station, TX). Two-sided p-values < 0.05 were considered statistically significant.

### Ethics

All methods were carried out in accordance with relevant guidelines and regulations. Informed consent was obtained from all individual participants included in the study. The ethics committee of the canton of Vaud, Switzerland, approved the conduct of Wave 8 and the conduct of the second SHARE Corona Survey (approval number 66/14).

## Data Availability

The datasets generated and/or analysed during the current study are available to the scientific community upon submitting a data requestion application to the SHARE study (https://share-eric.eu/data/become-a-user). Additional materials can be received upon request on: maud.wieczorek@unil.ch.
